# Novel Modulators of Proteostasis: RNAi Screen of Chromosome I in a Heat Stress Paradigm in *C. elegans*

**DOI:** 10.3390/cells7060049

**Published:** 2018-05-26

**Authors:** Andreas Kern, Natalie Spang, Heike Huesmann, Christian Behl

**Affiliations:** Institute of Pathobiochemistry, University Medical Center of the Johannes Gutenberg University, Duesbergweg 6, 55099 Mainz, Germany; akern@uni-mainz.de (A.K.); spang.natalie@web.de (N.S.); duerk@uni-mainz.de (H.H.)

**Keywords:** *C. elegans*, RNAi screen, proteostasis, proteostasis network, autophagy, UPS, chaperone, neurodegeneration

## Abstract

Proteostasis is of vital importance for cellular function and it is challenged upon exposure to acute or chronic insults during neurodegeneration and aging. The proteostasis network is relevant for the maintenance of proteome integrity and mainly comprises molecular chaperones and two degradation pathways, namely, autophagy and the ubiquitin proteasome system. This network is characterized by an impressive functional interrelation and complexity, and occasionally novel factors are discovered that modulate proteostasis. Here, we present an RNAi screen in *C. elegans*, which aimed to identify modulators of proteostasis in a heat stress paradigm. The screen comprised genes that are located on chromosome I of the nematode and has identified 185 genetic modifiers, whose knockdown has enhanced the misfolding of a reporter protein upon temperature increase. Subsequently, we evaluated the effect of a distinct number of the identified candidates in an additional *C. elegans* model strain, which expresses the aggregation-prone PolyQ35::YFP protein. Moreover, we annotated the human orthologs of the identified proteins and analyzed their enrichment in functional clusters and, as appropriate, their association with human neuropathologies. The achieved data collection includes several factors that have already been functionally associated with the proteostasis network, which highlights the potential of this heat stress-based proteostasis screen in order to detect novel modulators of proteome integrity.

## 1. Introduction

The integrity of the cellular proteome, which is referred to as protein homeostasis or proteostasis, is regularly challenged and proteins generally belong to the most skilled but instable components of the cell [1]. Under stress conditions, such as oxidative stress or increased temperatures, even stably folded proteins might experience conformational changes, and the increased appearance of misfolded or aggregated proteins causes cellular dysfunction [2,3]. Several diseases, including neurodegenerative disorders, as well as aging, are characterized by the accumulation of misfolded and aggregated proteins, which are directly related to a decline in the proteostasis network function [4,5,6]. This network assembles factors that are relevant for the maintenance of proteome integrity and comprises molecular chaperones and the two major cellular degradation pathways, autophagy and the ubiquitin proteasome system (UPS) [7,8]. These systems are interrelated and operate highly interactively during the entire lifetime of a protein, so as to support protein folding and assembly after synthesis, refolding of damaged proteins, or transfer to degradation pathways. Each stage is linked by the activity of molecular chaperones that, in combination with co-chaperone regulators, decide whether a misfolded protein is stabilized and refolded or ultimately degraded [3,9]. One of these co-chaperones is BAG3, which directs the chaperone HSC70 to protein clearance via autophagy and simultaneously provokes a decrease in the proteasomal protein degradation, which indicates a potential cross talk between the two basic degrading systems [10,11,12]. This example demonstrates the extensive functional conjunction of proteostasis factors, which is also represented by the complexity of the network that might comprise up to 2000 proteins [3]. To date, several large-scale genetic screens have been performed to elucidate novel factors that modulate proteostasis, employing, for example, *C. elegans* model systems, which overexpress disease-related aggregation-prone proteins [13,14,15,16].

Here, we present an RNAi screen in the nematode, which has aimed to identify modulators of proteostasis, specifically in a heat stress paradigm. Our approach was to detect the cellular factors whose knockdown enhances the misfolding of the cytosolic reporter protein Luciferase::GFP (LUC::GFP), which is expressed in the muscle cells of the worm [17], upon increased temperatures. The feeding RNAi screen covered all of the genes that are located on chromosome I and reproducibly identified 185 genetic modifiers in total. These candidates might directly or indirectly influence the capacity of the cellular proteostasis network and/or proteostasis. Additionally, we evaluated many of the identified candidates for their impact on protein aggregation in a *C. elegans* model strain expressing the aggregation-prone PolyQ35::YFP protein. Moreover, we annotated the human orthologs of the identified factors and analyzed their enrichment in functional clusters, as well as their association with human neuropathologies, when applicable. The achieved data collection includes several novel but also known factors that have already been functionally linked to proteostasis, which demonstrates the potential of this *C. elegans* heat stress-based RNAi screen. 

## 2. Material and Methods

### 2.1. C. elegans Strains and Maintenance

According to standard procedures, *C. elegans* were maintained at 20 °C on nematode growth medium (NGM) plates that were seeded with HB101 *E. coli*. The following strains were employed in this study: P*_myo-_*_3_::LUC::GFP/pRF4, expressing LUC::GFP in the body wall muscle cells [17]; and the strain AM140 (rmls126 [P*_unc-_*_54_::Q35::YFP]), expressing PolyQ35::YFP in muscle cells [18]. The latter strain was obtained from the *Caenorhabditis* Genetic Center (USA). 

### 2.2. RNA Interference Screen

The RNAi screen for the identification of factors of proteostasis in the muscle cells of *C. elegans* was performed using the commercially available RNAi feeding library that is equipped with HT115 *E. coli* expressing dsRNA, for approximately 85% of the predicted *C. elegans* genes (Source BioScience, Nottingham, UK), which was generated by the group of Julie Ahringer [19]. The RNAi bacteria were grown to an optical density of OD_600_ = 0.5 in LB medium/ampicillin (50 µg/ ml) at 37 °C with continuous shaking and were plated on RNAi plates, which consisted of NGM that was supplemented with 1 mM β-D-isothiogalactopyranoside to induce dsRNA synthesis and 50 µg/ml ampicillin. In order to obtain an age-synchronized population of worms, L4 larvae (the last larval stage before adulthood) of the P*_myo-_*_3_::LUC::GFP strain were transferred onto RNAi plates. After 24 h, the adult worms were put onto fresh plates and were allowed to lay eggs for around 1 h. Afterwards, the adult worms were discarded, which resulted in an age-synchronized population of progeny. These nematodes were maintained on the RNAi plates at 20 °C for 72 h. Subsequently, they were heat stressed at 38 °C (±0.4 °C) on RNAi plates in an incubator for approximately 1 h. The influence of the knockdown of the library clones on LUC::GFP was analyzed once GFP punctae of the reporter protein were observed in the worms that were treated with hsp-110 RNAi as positive control (for a detailed description, see Results and Discussion). The worms that were treated with empty vector carrying bacteria served as the negative control, and the experiment was only deemed valid when these worms showed no LUC::GFP accumulations, but the hsp-110 RNAi-treated nematodes were positive. The effect of each RNAi treatment was analyzed in 10 to 15 worms and was evaluated as positive when at least 50% of the worms showed an enhanced LUC::GFP accumulation. The encoded genes of the library clones were unknown to the investigator and were annotated subsequently to the screening. The worms showing severe phenotypes during the period of RNAi treatment (e.g., larval arrest or lethality) were excluded from the analysis. The visual inspection of LUC::GFP was conducted using a fluorescence stereomicroscope (Leica, Wetzlar, Germany).

### 2.3. PolyQ35::YFP Aggregation

To analyze the effect of the candidates that were identified in the RNAi screen on proteostasis in a model system that expresses aggregation-prone proteins, we utilized the AM140 strain that expresses PolyQ35::YFP in the muscle cells [15,18]. The RNAi-mediated knockdown of the respective factors was performed exactly as it is described for the RNAi screen, and an investigator that was blinded to the condition quantified the total number of PolyQ35::YFP aggregates by visual inspection, using the fluorescence stereomicroscope.

### 2.4. Data Analysis

The identified candidates were analyzed on www.wormbase.org. WormBase is set up by an international consortium of biologists and computer scientists that are dedicated to provide accurate, current, and accessible information concerning the genetics, genomics, and biology of *C. elegans*. The bioinformatics resource DAVID (https://david.ncifcrf.gov/; [20,21]) was used to determine the enrichments of the human orthologs in functional clusters. The clusters with *p* values of ≤0.05 were selected as significant. The disease-association of human orthologs was annotated employing www.ensembl.org and www.uniprot.org.

## 3. Results and Discussion

Previously, we generated a worm strain that expresses cytosolic LUC::GFP in the body wall muscle cells and in subsequent studies, we showed that the correct conformation of the reporter protein is influenced by the functional capacity of the cellular proteostasis network [17]. Alterations in the network activity that were mediated, for example, by a knockdown of the heat shock transcription factor 1 (*hsf-1* RNAi), resulted in an increased misfolding and accumulation of LUC::GFP upon heat stress, when compared to the empty vector-treated control worms. When the worms were allowed to recover at normal cultivation temperature subsequent to the heat stress, the reporter protein was efficiently refolded and GFP-positive punctae completely dissipated [17]. Importantly, the expression of LUC::GFP alone showed no detectable phenotypes in the muscle cells and it did not influence the worm development or behavior in general.

**Figure 1:**
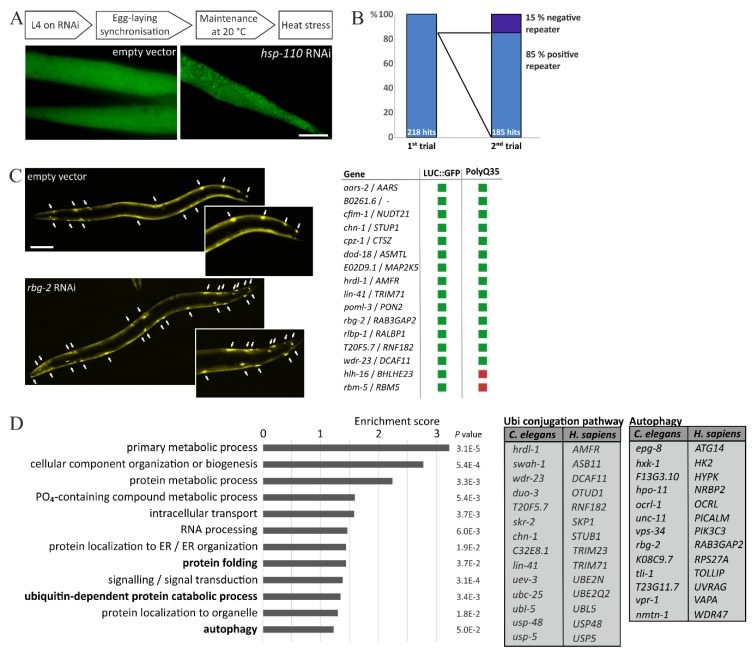
Evaluation of the heat stress-based RNAi screen. (**A**) Schematic sequence of the RNAi approach and representative fluorescence images of body wall muscle cells of LUC::GFP-expressing worms after heat stress and the respective RNAi treatment. Scale bar: 20 µm. (**B**) Statistics of first and second trial. Initially, 218 candidates were identified, of which 85 % repeated their effects on LUC::GFP upon heat stress. (**C**) Representative fluorescence images of PolyQ35::YFP after RNAi treatment. Arrows indicate aggregates. Scale bar: 100 µm. The table summarizes candidates that were evaluated for their impact on PolyQ35::YFP aggregation. Green square: increased aggregation; Red square: no impact on aggregation. (**D**) Human orthologs of identified candidates were analyzed for functional clusters employing the bioinformatics tool DAVID. The tables depict candidates functionally associated with the ubiquitin conjugation pathway or autophagy, respectively.

By employing this model system, we conducted an RNAi screen and knocked down about 2875 genes that are located on the chromosome I of *C. elegans* to analyze their individual impact on LUC::GFP upon temperature increase. The knockdown of each single gene was carried out by feeding RNAi, using the commercial RNAi library [19], which is frequently used for large-scale RNAi approaches [14,15,19,22]. We established the RNAi-mediated knockdown of hsp-110 as a positive control for the screening of the library clones, since we observed that a deficiency of this chaperone reliably enhanced the formation of LUC::GFP punctae, during increased temperatures. Thus, after the RNAi treatment, the worms were moved into heat stress conditions and as soon as we observed LUC::GFP accumulations in body wall muscle cells of the hsp-110 RNAi treated worms, we analyzed the performance of the reporter protein in the library clones (Figure 1A). With this approach, we identified 185 genetic modifiers of proteostasis, whose knockdown had resulted in enhanced accumulations of LUC::GFP during heat stress (complete list of candidates see Supplementary Materials Table S1). This number corresponds to approximately 6.5% of the total quantity of screened clones. All of the presented candidates were confirmed for their impact on LUC::GFP in a second screening round and, importantly, 85% of the candidates that were identified in the first trial also replicated their effect in a second screen (Figure 1B). This underlines the stringency of our approach. Additionally, we re-evaluated 90 randomly picked genes that did not affect LUC::GFP in the first round and, indeed, these clones demonstrated no influence on the reporter protein in further trials. 

Importantly, in the chosen approach, the knockdown of a respective gene was conducted in an otherwise healthy nematode and LUC::GFP misfolding was induced by increased temperatures. Therefore, in this particular paradigm, the proteostasis was challenged upon heat stress, but not during the worm development, as, for example, triggered by the time-dependent aggregation of aggregation-prone proteins. Indeed, in most of the screened clones, the RNAi-mediated knockdown showed no phenotypes during the period of the RNAi treatment, but exclusively accelerated the appearance of the LUC::GFP accumulations during heat stress. This is exemplified by the knockdown of hsp-110, which exhibited no detectable alterations in the worm, but resulted in enhanced GFP-positive accumulations after the application of the secondary stress. However, the identified candidates might have directly or indirectly affected the stability of LUC::GFP, by either being a direct functional component of the proteostasis network or by indirectly challenging proteostasis, because of the detrimental effects of their deficiency on alternative cellular functions that negatively reflected on protein homeostasis.

To further confirm the impact of the identified candidates on cellular proteostasis, we selected a distinct number and analyzed their influence on the protein aggregation in a worm model that expresses the aggregation-prone PolyQ35::YFP protein. This strain, which is characterized by a time-dependent aggregation of PolyQ35::YFP and is frequently used as an in vivo model for PolyQ/Huntington disease, had already been employed in genome-wide RNAi screens for the identification of novel genetic modifiers of proteostasis [15,16,18]. 

Indeed, the RNAi-mediated knockdown of most of our chosen candidates aggravated the aggregation of PolyQ35::YFP and resulted in a substantially increased number of YFP-positive punctae (Figure 1C). This observation augmented the influence of these particular candidates on cellular proteostasis. However, the knockdown of two of the selected factors did not affect the PolyQ35::YFP aggregation, but it repeatedly resulted in enhanced accumulations of LUC::GFP, upon heat stress. This illustrates that both approaches, namely, heat stress and PolyQ35::YFP aggregation, differentially select candidates but, obviously, also demonstrates that each factor has to be confirmed in adequate additional model systems for its particular impact on proteome integrity. 

In subsequent studies, we already analyzed the candidate RBG-2/RAB3GAP2 and successively also analyzed the enzymatically active RBG-1/RAB3GAP1, which form the heterodimeric RAB3GAP complex, for their detailed impact on proteostasis. Employing *C. elegans* model systems as well as human cell lines, we showed that the RAB3GAPs potently modulate autophagy and, interestingly, act on the degradative pathway in collaboration with the RAB GTPase RAB18, to directly affect the protein aggregation and proteostasis [23,24]. Thus, originating from the RNAi screen that was presented here, we already characterized RBG-1/RBG-2 and could transfer their impact on autophagy and proteostasis into the human system. 

Importantly, a significant number of *C. elegans* genes displays orthologs in humans and we examined the identified candidates for the existence of human orthologs, using appropriate online resources. Indeed, approximately 77% of the identified modulators of proteostasis have genetic counterparts in humans and, subsequently, we analyzed their enrichment in functional clusters (Figure 1D), employing the bioinformatics resource DAVID [20,21]. The identified clusters do not allow a statistical conclusion with regard to the whole genome, since we exclusively screened the genes that are located on chromosome I. However, three of these functional clusters can be directly linked to the maintenance of proteostasis, namely: protein folding, ubiquitin-dependent protein catabolic process, and autophagy. This underlines the potential of our RNAi screen, and the candidates that are associated with the ubiquitin conjugation pathway and autophagy have been listed in Figure 1C. Importantly, it was recently demonstrated that autophagy is up-regulated by mild heat stress and HSF-1 overexpression [25], which highlights the correlation of increased temperatures, autophagy, and proteostasis, which is also indicated by our identified candidates. 

**Figure 2:**
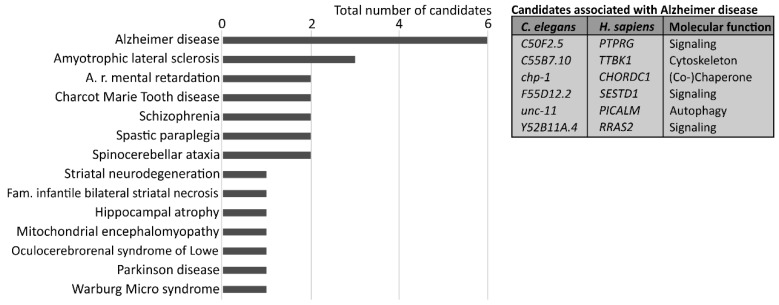
Association of identified candidates with human neuropathologies. Human orthologs were linked to neuropathologies employing online resources and are represented relative to their total number. The table lists identified candidates related to Alzheimer disease

Consequently, we also evaluated the human orthologs for a potential association with human diseases and focused on neuropathologies (Figure 2). Again, this analysis does not represent any statistical assumption. Interestingly, many of the identified factors are linked to human neuropathologies and several of the neurological disorders are indeed related to increased protein misfolding and aggregation [26,27]. Six modifiers of proteostasis that were identified in our RNAi approach are associated with Alzheimer disease (Figure 2), which is the most frequent form of dementia in humans, and shows an age-related neurodegeneration. Importantly, although the exact causative pathway of sporadic Alzheimer disease is still unknown [28,29], the disorder has been associated with the accumulation of intra- and extra-cellular aggregates and a decline in proteostasis network function. Several genome-wide association studies identified genetic risk factors for Alzheimer disease and, interestingly, the single nucleotide polymorphisms in the gene coding for PICALM (phosphatidylinositol binding clathrin assembly protein) were linked to the dementia [30]. PICALM, which was also found in our RNAi screen, modulates autophagy and its activity is important for the autophagic degradation of Alzheimer disease-related proteins [31,32,33], which highlights the association of proteostasis network function, proteome integrity, and disease.

Collectively, here we have presented a heat stress-based large-scale RNAi screen and additional successive analyses to identify and characterize candidates that modulate the proteome integrity in *C. elegans*. Our studies investigating the role of RBG-1/RBG-2 in proteostasis have already highlighted the high mechanistic capacity and translational potential of this approach [23], which provides multiple promising factors that are important for the maintenance of proteostasis, and might be relevant for the pathological mechanisms underlying human neurodegenerative diseases, as well as aging. 

## Supplementary Materials

Supplementary materials are available on http://www.mdpi.com/2073-4409/7/6/49/s1.
